# Machine learning-based techniques to improve lung transplantation outcomes and complications: a systematic review

**DOI:** 10.1186/s12874-022-01823-2

**Published:** 2022-12-23

**Authors:** Marsa Gholamzadeh, Hamidreza Abtahi, Reza Safdari

**Affiliations:** 1 Health Information Management Department, School of Allied Medical Sciences, Tehran University of Medical Sciences, 5th Floor, Fardanesh Alley, Qods Ave, Tehran, Iran; 2 Pulmonary and Critical Care Medicine Department, Thoracic Research Center, Imam Khomeini Hospital Complex, Tehran University of Medical Sciences, Tehran, Iran

**Keywords:** Lung transplantation, Machine learning, Review, Lung diseases

## Abstract

**Background:**

Machine learning has been used to develop predictive models to support clinicians in making better and more reliable decisions. The high volume of collected data in the lung transplant process makes it possible to extract hidden patterns by applying machine learning methods. Our study aims to investigate the application of machine learning methods in lung transplantation.

**Method:**

A systematic search was conducted in five electronic databases from January 2000 to June 2022. Then, the title, abstracts, and full text of extracted articles were screened based on the PRISMA checklist. Then, eligible articles were selected according to inclusion criteria. The information regarding developed models was extracted from reviewed articles using a data extraction sheet.

**Results:**

Searches yielded 414 citations. Of them, 136 studies were excluded after the title and abstract screening. Finally, 16 articles were determined as eligible studies that met our inclusion criteria. The objectives of eligible articles are classified into eight main categories. The applied machine learning methods include the Support vector machine (SVM) (*n* = 5, 31.25%) technique, logistic regression (*n* = 4, 25%), Random Forests (RF) (*n* = 4, 25%), Bayesian network (BN) (*n* = 3, 18.75%), linear regression (LR) (*n* = 3, 18.75%), Decision Tree (DT) (*n* = 3, 18.75%), neural networks (*n* = 3, 18.75%), Markov Model (*n* = 1, 6.25%), KNN (*n* = 1, 6.25%), K-means (*n* = 1, 6.25%), Gradient Boosting trees (XGBoost) (*n* = 1, 6.25%), and Convolutional Neural Network (CNN) (*n* = 1, 6.25%). Most studies (*n* = 11) employed more than one machine learning technique or combination of different techniques to make their models. The data obtained from pulmonary function tests were the most used as input variables in predictive model development. Most studies (*n* = 10) used only post-transplant patient information to develop their models. Also, UNOS was recognized as the most desirable data source in the reviewed articles. In most cases, clinicians succeeded to predict acute diseases incidence after lung transplantation (*n* = 4) or estimate survival rate (*n* = 4) by developing machine learning models.

**Conclusion:**

The outcomes of these developed prediction models could aid clinicians to make better and more reliable decisions by extracting new knowledge from the huge volume of lung transplantation data.

**Supplementary Information:**

The online version contains supplementary material available at 10.1186/s12874-022-01823-2.

## Introduction

The last decade has seen a gradual but significant increase in organ transplants, although lung transplants have a lower rate than other organ transplants [1]. Lung transplantation (LTx) is a well-established treatment for a wide variety of end-stage lung diseases [2, 3]. By 2019, more than 4,500 lung transplants were executed at 260 centers worldwide, according to the International Heart and Lung Transplant Association (ISHLT) [4]. Despite all advances in medicine, LTx encounters some difficulties like lung allograft dysfunction, organ rejection, side effects of immunosuppressive therapy, or complex infection yet [5]. Nowadays, predicting transplant complications, determining risk factors, and estimating the success rate of transplants are the main concerns of physicians in this field with the increase in transplant cases. Developing prediction models could aid clinicians in organ allocation and estimating post-transplantation outcomes and complications [6]. Though traditional statistical methods have not been able to meet their needs, some predictive models were developed to estimate the post-transplant survival rate or side effects with the hope of increasing the success of transplantation using Artificial Intelligence (AI) or machine learning (ML) techniques.

It seems that the development of data-driven ML techniques can support clinicians in making more informed decisions by generating new insights into disease in medicine [7]. Machine learning (ML) techniques are a set of methods for analyzing a large amount of data to reveal hidden patterns in data sets or explain the relationship between various variables [8]. Machine learning methods in medicine have been applied in a wide range of areas such as cancer problem-solving, medicinal chemistry, brain and neurology, medical imaging, and data analysis of wearable sensors for symptom monitoring [9]. ML methods can deal with large and complex medical data and analyze them easily to find new ways for accurate diagnosis and treating patients [10, 11]. Utilizing ML models in other organ transplants could estimate the risk of acute rejection, survival rate, risk factors, and prevalent comorbidities after transplantation [12]. In addition, they can determine the most appropriate organ recipient and those at high risk of post-transplant mortality by building ML models based on various parameters [13].

Many efforts have been made to develop predictive models in LTx using machine learning techniques [13–15]. No study has been published to investigate the applied methods in this domain. Our study aims to systematically review all published evidence on the utilization of ML techniques as one of the main approaches of artificial intelligence in lung transplantation. In addition to providing a comprehensive overview of the most widely used machine learning methods in LTx, our additional objectives include identifying the main challenges and concerns that machine learning methods are being built to deal with them.

## Method

### Research questions

This study was conducted to answer the following research questions:

R-Q1- Which machine learning techniques are used in the lung transplantation domain?

R-Q2- For which specific problems and objectives in lung transplantation has machine learning been used?

R-Q3- In which stages of lung transplantation have machine learning methods been applied?

R-Q4- What data sources or databases have been used in lung transplantation to develop machine-learning models?

RQ-5- Which features are used by the machine learning models reviewed studies?

RQ-6-Which evaluation techniques have been employed in developed models?

## Search strategy

A systematic search was conducted in six electronic databases, Medline (PubMed), Scopus, Web of Science (WOS), PsycINFO, IEEE, and Cochrane databases using pre-specified search strategies based on keywords. Database searching was performed in the period from January 2000 to June 2022. Reference lists of retrieved articles and review articles in the field were also searched to identify eligible studies that met inclusion and exclusion criteria. This systematic review was conducted using the Preferred Reporting Items for Systematic Reviews and Meta-Analysis (PRISMA) checklist [16].

## Inclusion and exclusion criteria

Our research questions were outlined based on PCC (Population, Concept, Context) to conduct qualitative review studies [17]. Population refers to lung transplant recipients, candidates for lung transplantation, or those on the waiting list. The concept referred to lung transplantation and all related complications, outcomes, side effects, and affective factors. Context referred to any machine learning techniques applied in LTx.

We included all full-text articles focusing on the utilization of ML techniques in lung transplantation. The inclusion criteria for this review were as follows: (1) original research study, (2) Studies to be included if it has provided sufficient information on the machine learning algorithms used for the analysis, (3) Article is included if it evaluated the applied ML techniques, (4) Topics related to lung transplantation, (4) All types of lung transplant recipients (single or double), candidates, or patients in waiting list.

Non-peer-reviewed articles, all types of review articles, meta-analyses, letters to the editor, commentaries, abstracts, editorials, patents, perspectives, or studies with non-human species were excluded. Studies were excluded too if they (1) were not full-length publications, (2) The protocol or methods papers, (3) Not English papers, and (4) Machine Learning methods were not used at all.

## Data extraction process

Two researchers screened the title and abstracts of extracted articles, independently. The screening process was done using the PRISMA checklist. Then, two researchers (MG and RS) read the full texts. The disagreement was resolved by the supervision of the other researcher (HA).

The details of the methodology and outcomes of reviewed articles were noted on a data extraction sheet. The data extraction process was done by two reviewers. Data elements extracted included title, publication year, country, population, feature selection technique, input variables, applied ML method, training and validation size, validation techniques, and model performance indicators.

## Risk of bias

The risk of bias in included articles was evaluated by two independent reviewers using the Cochrane collaboration risk of bias tool suggested by Narukab [14] for ML-related articles. The methodological quality of the articles was assessed based on these domains: (1) Data collection, (2) Study Response, (3) Outcome Measurement, (4) and Statistical Analysis and Reporting. The high risk, not clear, and low risk of bias was allocated to each study.

## Quality assessment

Due to the heterogeneity of reviewed studies, the quantitative analysis was inappropriate. Hence, the quality of reviewed articles was evaluated by a quality assessment table for machine learning studies suggested by Qiao [18]. According to Qiao’s study, articles in the machine learning field are reviewed in nine areas in terms of quality. These categories include limits in the current non-machine learning approach, valid methods for over-fitting, predictors for an explanation, hyperparameters, using external data validation, feature engineering methods, applied platforms, stability of results, and suggested clinical use.

## Results

**Fig. 1:**
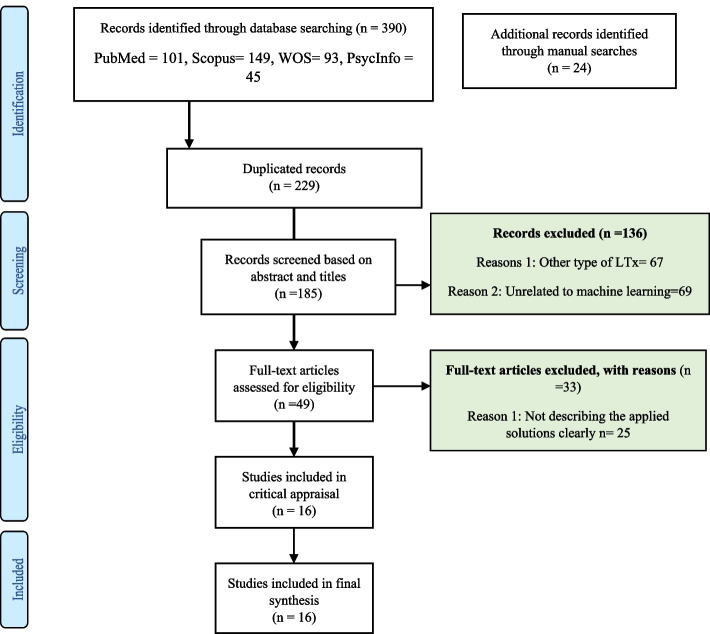
The flow diagram of article screening according to the PRISMA checklist

Searches yielded 414 citations. Of them, 185 articles remained after duplication removal. From 185 retrieved articles, 136 studies were excluded after the title and abstract screening because their topics were inapplicable to our subject. Next, the full text of 49 articles was reviewed according to inclusion criteria. Finally, 16 articles were determined as eligible studies that met our inclusion criteria. The screening process and the reasons for deleting articles following the PRISMA report checklist are described in Fig. 1.

Of 16 studies, 12 (75%) of the articles were published after 2015 [13, 19–28]. In terms of country, ten studies were conducted in the USA [13, 20, 22, 25, 28–33], and one study each in Belgium [21], China [27], Iran [19], Italy [23], Spain [24], and UK [26]. The summaries of applied techniques and characteristics of articles are described in Table 1.

**Table 1:** Summaries of reviewed articles

Author	Year	Objective	Population	Data source	Number of inputs in the final model	ML method	Validation method	Model performance	Result
Troiani and Carlin [29]	2004	Predict the incidence of disease after transplantation	30 subjects	A database of home monitoring data	Six different ordinals symptom measures	Bayesian models, Markov chain Monte Carlo (MCMC) methods	Cross-validation	For the Bayesian model: ROC curve < 0.78, Sensitivity = 71.5, Specificity = 91.3	Bayesian models have the best performance in comparison with the Markov model.
Oztekin.A et al. [30]	2009	Predicting the graft survival	16,604 cases	UNOS	283 variables	decision trees, neural networks, logistic regression, Cox regression models	Ten-fold cross-validation, Confusion matrix, Sensitivity, Specificity, Accuracy	The accuracy ranged from 78–86% for logistic regression, from 79–86% for neural networks, and from71–79% for decision trees	The undiscovered relationships were founded among the survival-related variables.
Delen.D et al. [31]	2010	Predict the risk factors for transplantation	310,773 records and 565 variables	UNOS	14 variables	SVM, ANN, MLP, RBF, DT (M5, CART), K-means	MSE, R2, 10-fold cross-validation, sensitivity analysis	SVM with an R2 value of 0.879, Neural network with an R2 value of 0.847 M5 algorithm-based regression tree model with R2 value of 0.785.	Thoracic organ recipients could be classified into ‘‘three’’ risk groups, namely low, medium, and high using a k-means clustering algorithm.
Oztekin.A et al. [32]	2011	Predicting the performance of patients after transplantation	16,771 records and 442 variables.	UNOS	27 variables	Bayesian neural networks	10-fold cross-validation, the R2 value of 0.68	The R2 value of 0.73	The ML models are superior to the existing techniques in terms of both prediction and interpretation capabilities.
Hosseini-Baharanchi. F et al. [19]	2016	Predict Bronchiolitis Obliterans Syndrome incidence	44 LTX recipients who survived ≥ 3 months post-LTX	Masih Daneshvari Hospital database	Five variables	Bayesian non-parametric model	Hazard ratio (HR), Monte Carlo error (MC-error)	MC-error values lower than 0.01	Our analysis revealed that CMV infection was associated with a significant increase in the risk of developing BOS.
Pande.A et al. [20]	2017	Predict pulmonary functions after transplantation	9471 FEV1 evaluations were available from 509 LTx patients	Cleveland Clinic data	17 variables	Gradient Boosting, generalized additive models (GAM), Boosted multivariate trees for spirometry data	cross-validation method	Standardized RMSE (sRMSE) averaged over 100 independent replications.	Developed models illustrate that forced 1-second lung expiratory volume (FEV1) has an important feature-time interaction for lung transplant patients.
Barbosa.E et al. [33]	2017	Predict Bronchiolitis Obliterans Syndrome incidence	176 LTx patients	Cardiothoracic clinic	The predictors were qCT metrics, PFTs, or SQS.	Multivariate logistic regression, SVM	The model’s prediction performance was assessed by AUC or Area under a ROC curve in cross-validated samples	Combination of MVLR and SVM based on PFT values: Max AUC 0.771, whereas models using qCT metrics-only outperformed models: max AUC 0.817 SVM models utilizing PC from qCT outperformed PFT (AUC = 0.817 vs. AUC = 0.767),	Combinations of qCT metrics with PFTs could predict BOS in the LTx group
Oztekin.A et al. [22]	2018	Predict quality of life	60,888 records and 443 features	UNOS	147 input features	Genetic algorithm, GA-kNN, GA-ANN, and GA-SVM models	5-fold cross-validation	Precisionclass1 = 0.992, Sensitivityclass1 = 0.998, Specificity Class1 = 0.996, F-Measure Class1 = 0.995, And G-Meanclass1 = 0.994, AUC = 85%	Applying GA-ANN, GA-kNN, and GA-SVM models proved that the performance of the lung transplantation process could be improved by the GASVM approach.
Barbosa.E. et al. [21]	2018	Predict Bronchiolitis Obliterans Syndrome incidence	71 LTx patients	Belgium clinic	14 variables	Support vector machines (SVMs)	R2 score	Accuracies for SVM: 83%. Sensitivity of 73.3% and a specificity of 92.3%	ML utilization showed that qCT metrics predict the eventual onset of BOS.
Mark.E et al. [13]	2019	Predict survival rate	20 000 samples	UNOS	128 variables	Linear regression, Cox proportional hazards model, Random Forest	10-fold cross-validation	RMSE = 5.4, 9.0, and 5.3 for the heart, liver, and lung recipients	For all investigated organs, five-year survival was predicted for the majority of patients.
Braccioni.F et al. [23]	2020	The role of CPET parameters in the development of respiratory symptoms in lung recipients	Twenty-four bilateral LTx recipients.	Tertiary teaching Hospital in Northeast Italy	Nine variables	Forest-Tree as ensemble-of-trees methods	5-fold cross-validation	Bottom boxes, correlation matrix, coefficient score, and box plot for each split.	Muscle pain at peak exercise was associated with basal and exercise- metabolic altered pathways. Dyspnea was associated with the intensity of ventilatory response
Dueñas-Jurado.J et al. [24]	2021	Predict recipient-donor matching	404 lung transplants	Reina Sofı´a University Hospital	48 variables	logistic regression (LR), product unit neural networks (PUNNs)	10-fold cross-validation	Chi-Square, coefficient score, and correlation were investigated to estimate the developed models	The proposed models represent a powerful tool for donor-recipient matching.
Shaish.H et al. [25]	2021	Predict survival rate	221 CT images of ILD patients	Institutional radiology database	Five variables	CNN, univariable logistic regression model, Cox regression analysis	Five-fold cross-validation	AUC = 0.7417, Sensitivity = 77%, Specificity = 66% for the CNN model	Virtual lung wedge resection in patients with ILD can be used as an input to a CNN for predicting the histopathologic UIP pattern and transplant-free survival.
Zafar.F et al. [28]	2022	Predict recipient-donor matching	19,263 eligible double LTxs	UNOS	43 variables	LASSO Cox regression, Random Forest tree, COX regression	Not mentioned	The covariate levels of each recipient and the adjusted total risk score was computed for every recipient and the density plot	LAPT could be effective in matching donor-recipient through lung transplantation.
Su.J et al. [27]	2022	Determine the role of infection in rejection	181 sputum samples from 59 L	Guangzhou Medical University	34 variables	Random Forest models	10-fold cross-validation	AUC for the combination of procalcitonin (PCT), the six-genera, and T-lymphocyte levels were 0.919, 0.898, and 0.895	Airway microbiota along with PCT and T lymphocyte levels were determined as predictive factors in infection and acute rejection.
Stefanuto.P et al. [26]	2022	Predict primary graft dysfunction after lung transplantation	35 lung transplants recipients	Harefield Hospital	27 variables	Support vector machine (SVM) with a linear kernel, Multivariate analysis of variance (MANOVA)	Not mentioned	For SVM, AUROC = 0.90 and an accuracy of 0.83	Three main chemical classes that were effective in PGD prediction were identified using model development.

## machine learning methods applied in reviewed articles

Among 16 papers, the Support vector machine (SVM) technique (*n* = 5, 31.25%) is the most favorable method utilized by the authors [21, 22, 26, 31, 33]. After that, logistic regression (*n* = 4, 25%) [24, 25, 30, 33], and random forests (RF) (*n* = 4, 25%) [13, 23, 27, 28] have been the most used techniques to develop data-driven models concerning lung transplantation. Bayesian network (BN) (*n* = 3, 18.75%) [19, 29, 32], linear regression (LR) (*n* = 3, 18.75%) [13, 26, 28], Decision Tree (DT) (*n* = 3, 18.75%) [30, 31], neural networks (*n* = 3, 18.75%) [24, 30, 31], Markov Model (*n* = 1, 6.25%) [29], KNN (*n* = 1, 6.25%) [31], K-means (*n* = 1, 6.25%) [31], Gradient Boosting trees (XGBoost) (*n* = 1, 6.25%) [20], and Convolutional Neural Network (CNN) (*n* = 1, 6.25%) [25] were other strategies used to develop machine learning models in studies. Only one article employed a deep learning method for image processing using CT features [25].

A brief description of applied machine learning techniques is represented in Table 2. Most studies (*n* = 11) employed more than one machine learning technique or combination of different techniques to make their models [13, 20, 22–25, 28–31, 33].

**Table 2:** A summary of the machine learning methods employed in LTx

Algorithms	Description
Support vector machine (SVM)	Support Vector Machine or SVM is one of the most popular classification algorithms for creating the best decision line or boundary. Its objective is to find a hyperplane in N-dimensional space (N is the number of features) that distinctly classifies the data points.
Logistic Regression	Logistic regression is utilized to evaluate the association of independent (predictor) features with a binary dependent (outcome) feature.
Decision Tree	A decision tree uses a set of rules to classify and visualize numerical and categorical data. A Decision Tree is used to generate simple and logical rules.
Random Forests (RF)	A random forest classifier is a meta-estimator that fits many decision tree models under different samples of the data sets. RF employs the average of decision trees to improve the model’s prediction accuracy and control overfitting.
Bayesian network and Naïve Bayes	The Naive Bayes algorithm was developed based on the Bayes theorem assuming independence between each pair of features. This algorithm demands a small amount of training data to estimate the necessary parameters.
Neural Networks	Neural networks or artificial neural networks (ANN) are a type of artificial intelligence that can be used in medicine for early and more accurate diagnosis of diseases. They make it possible to distinguish patients from those who are healthy.
Markov Model	Markov models are often used to model the probabilities of different states and the transition rates between them. This method is generally used to detect patterns, and predict and learn statistics of sequential data.
K-means	The k-Means algorithm is a clustering algorithm used to predict the probability of disease based on medical data sets.
Gradient Boosting trees (XGBoost)	Gradient boosting is a machine learning algorithm where tree-based classifiers are trained to reinforce each other to achieve outstanding outcomes. This method differs from Random Forests (RF) where trees are learned sequentially based on the performance of all previous trees.
Convolutional Neural Network (CNN)	The CNN-based deep neural system is widely used in the medical classification task. CNN is an excellent feature extractor to classify medical images to overcome complicated and expensive feature engineering.
KNN	K-Nearest-Neighbors (KNN) is one of the successful data mining techniques used in classification problems that refers to the number of nearest neighbors.

**Fig. 2:**
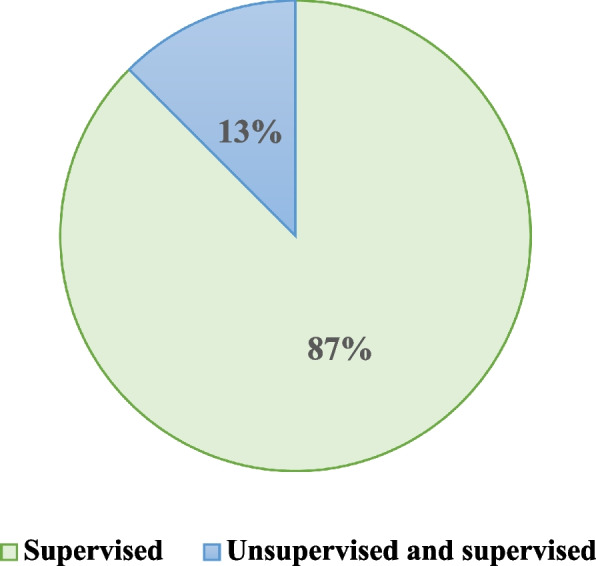
Proportion of various ML methods used in the literature

In all articles, ML methods can be divided into two broad categories, supervised and unsupervised learning techniques. Though in the machine learning field, there is another category for these techniques called transfer learning which was not employed in the reviewed studies. Most of the studies took only supervised machine learning techniques (Fig. 2). Only two studies have employed the combination of supervised and unsupervised learning techniques in reviewed articles.

## specific problems and objectives in lung transplantation

All studies applied ML methods with different approaches. The objectives of eligible articles are classified into eight main categories. All reviewed articles tried to solve some problems using machine learning techniques, these objectives and their frequency are described in Table 3. In most cases, developing ML models can help clinicians to predict acute diseases incidence after lung transplantation (*n* = 4) or estimate survival rate (*n* = 4).

**Table 3:** General characteristics of reviewed articles

Main objectives	Frequency	Percentage
Predict the acute disease events after transplantation	4	25.00%
Predict survival rate	4	25.00%
Predict recipient-donor matching	2	12.50%
Predict pulmonary functions/ pulmonary symptoms after transplantation	2	12.50%
Predict primary graft dysfunction after lung transplantation	1	6.25%
Determine the role of infection in rejection	1	6.25%
Predict the risk factors for transplantation	1	6.25%
Predict quality of life	1	6.25%

**Fig. 3:**
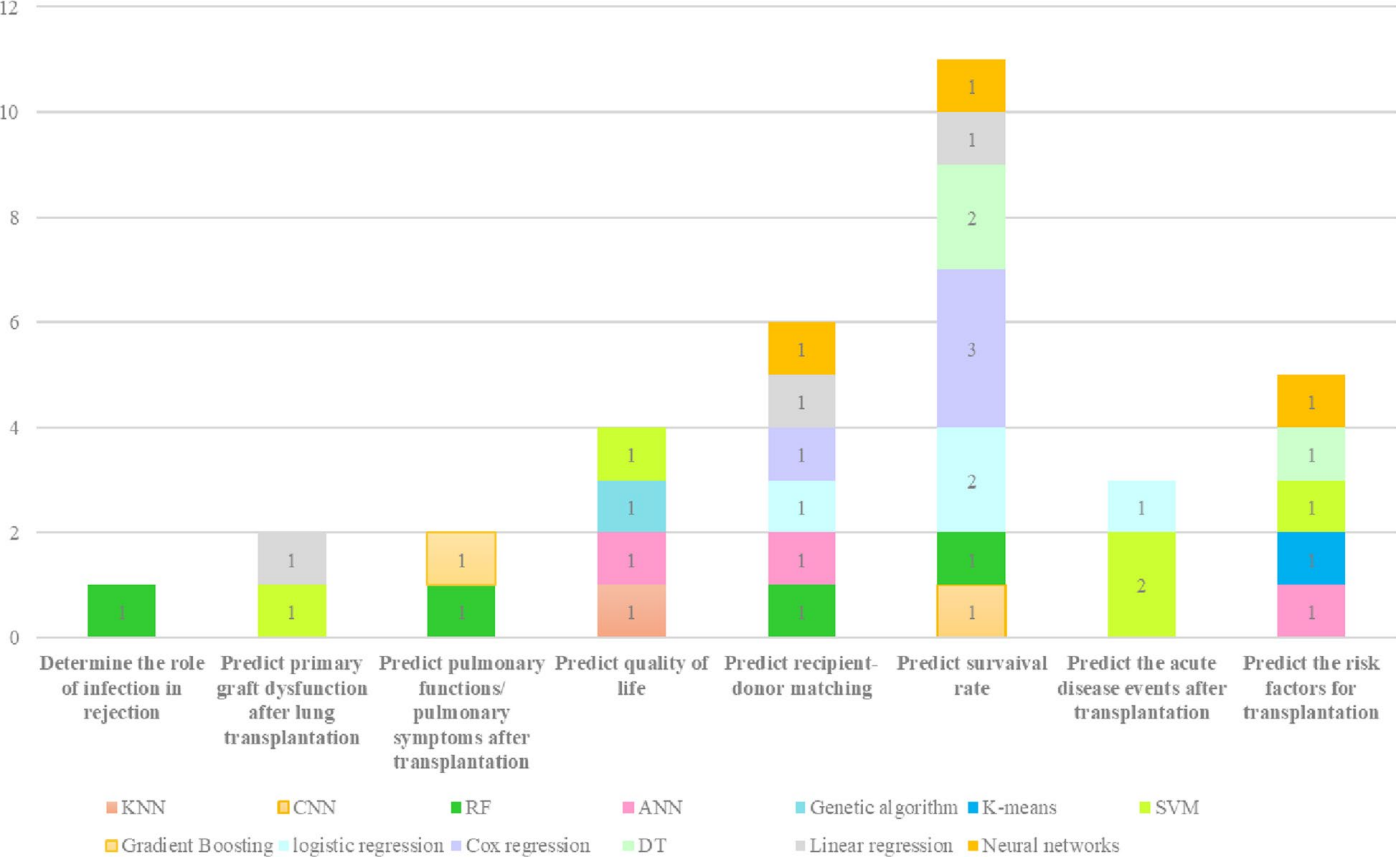
Distribution of ML methods in terms of their objectives

The distribution of applied ML techniques in terms of their objectives is represented in Fig. 3.

## Predicting the acute disease events after transplantation

The occurrence of some diseases after lung transplantation is inevitable. Evidence showed that more than 50% of lung recipients experienced bronchiolitis obliterans syndrome (BOS) development after transplantation surgery by five years [34]. Of 16 studies, four articles were devoted to improving the predicting BOS incidence after LTx. Troiani [29] and Hosseini-Baharanchi [19] applied the Bayesian network model to predict BOS incidence after LTx. Troiani [29] used spirometry variables and symptom covariates to predict BOS to compare different models. Their results showed that the Bayesian approach was preferable to classical approaches in BOS classification in lung transplant recipients. Similarly, Hosseini-Baharanchi [19] used demographic data and some clinical variables to predict BOS incidence after LTx. They found that CMV infection was a good predictor of BOS incidence in LTx patients.

Barbosa et al. [21, 33] developed classification models to predict BOS development using the SVM technique and logistic regression based on CT features in two studies. Their results showed that the combination of CT metrics with PFT (Pulmonary function tests) as predictors could enhance model diagnostic accuracy for all transplant types.

## Predicting the survival rate of post-transplantation

However, the survival rate of lung transplants compared to other organ transplants is low. But according to the International Society of Heart and Lung Transplantation (ISHLT) Report, the existing five-year survival rate after lung transplantation is approximately 55% [35]. Four articles are devoted to predicting and estimating the survival rate after LTx.

The results of the Oztekin.A et al. [30] the study showed using data-mining methodology could support clinicians to select more related variables which were effective in predicting graft survival. According to their study, the integration of neural network models, decision trees, and logistic regression with the Cox hazard model was able to show results with satisfactory prediction accuracy compared to the traditional methods that were used before.

Oztekin. A [32] and his collagenous in another study in 2011 succeed to develop a decision support tool using decision trees and neural networks. By designing this decision-making tool based on data mining models, the doctor in the case of organ donation can quickly decide which patient is the most suitable potential recipient for donor organ allocation.

Mark.E et al. [13] developed machine learning-based models to estimate 5-year survival rates for patients using linear regression and Random Forest. According to their models, patients are predicted to have a higher predicted survival accepting an increased risk for disease transmission (IRD) organ offer compared to waiting for a non–IRD organ with average wait times.

Shaish et al. [25] created his model using a deep learning method (CNN) to classify the patterns of disease progression for Usual interstitial pneumonia (UIP) patients and determine the severity of the disease with survival rate after LTx. Their results showed that CNN-predicted UIP was associated with an increased risk of death or lung transplantation during cross-validation.

## Predicting recipient-donor matching

Match-to-recipient (D/R) in lung transplantation (LTx) is usually determined based on blood group and predicted total lung capacity (pTLC), as well as height and age [36, 37]. Predicting the recipient-donor matching and predicting the most important factors could be beneficial for clinicians in selecting the most suitable recipient. Dueñas-Jurado [24] and Zafar. F [28] developed prediction models based on the characteristics of donor recipients and past experiences with lung donors and recipients to improve donor-recipient matching in lung transplantations.

Dueñas-Jurado et al. [24] created a model in the lung allocation system for matching lung transplant donor recipients using neural networks. The predictors used to predict the probability of survival rate and recipient-donor matching included lower pre-transplant carbon dioxide (PCO2) pressure, higher pre-transplant and post-transplant functional vital capacity (FVC), lower donor mechanical ventilation, and shorter ischemia time. The proposed model represented in this study was a powerful tool for donor-recipient matching that showed higher accuracy than classical statistical methods.

Similarly, Zafar. F [28] developed a comprehensive model to guide recipient-donor matching using random forest and cox regression using clinical and demographic data of recipients and donors. They developed an online Lung Transplantation Advanced Prediction Tool (LAPT) in the form of a simple calculator. Employing this tool, users can enter recipient and donor information to calculate predicted 1-, 5-, and 10-year survival, risk stratification, and associated survival and half-life predictions. Top selected common features that are effective in predicting quality of life and identified through this study included simultaneous lung, type of transplant, recipient CMV results at transplantation, recipient CMV results at transplantation, any drug-treated infection, chronic steroid at transplant, recipient age, prior cardiac surgery at transplant, and infection requiring IV drug therapy.

## Determining the relation between pulmonary function tests and LTx outcomes

Predicting Total Lung Capacity (pTLC) has a significant role in LTx outcomes. Hence, Pande. A et al. [20] explored the relationship between Forced expiratory volume in the first second (FEV1) and age of lung recipients with LTx status using the novel multivariate tree boosting method on longitudinal data of spirometry tests. Their investigation using FEV1 longitudinal data and application of the feature selection method revealed that double-lung recipients not only have a higher FEV1 but also have a slower decline in lung capacity than single-lung recipients. They succeeded to apply a novel multivariate tree-boosting method for fitting a semi-nonparametric model.

In another study, they investigated the role of cardiopulmonary exercise testing (CPET) parameters on symptoms of lung recipients after transplantation using a random forest tree [23]. Developing predictive models revealed that muscle pain at peak exercise was strongly associated with altered basal and exercise-induced metabolic pathways. The onset of dyspnea was associated with the intensity of the ventilatory response to meet the metabolic demands of increased workloads.

## Predicting the most important reasons for transplant rejection

Rejection is a major complication that remains an important problem after lung transplantation. Despite advances in immunosuppressive therapy and immunosuppressive drugs used, more than one-third of lung transplant recipients are treated for acute rejection in the first year after transplantation [38]. Some of the most important reasons for transplant rejection in patients are infection and primary graft dysfunction after lung transplantation. In this regard, Su. J et al. [27] utilized a random forest model to survey the association between airway infection and rejection in lung transplant recipients (LTRs). Developing models revealed the role of airway microbiota, especially together with PCT and T-lymphocyte levels in differentiating between clinically stable recipients and those with infection and acute rejection.

Stefanuto.P et al. [26] made prediction models using SVM techniques and multivariant analysis to discover the relation of pulmonary volatile organic compound (VOC) spectrum with primary graft dysfunction in lung transplant recipients. The authors succeeded to develop a model which identified patients with grade 3 primary graft dysfunction (PGD) with an AUROC of 0.90 and a positive predictive value of 0.88. This indefinable molecular approach represents a new molecular strategy for detecting and monitoring allograft injury.

## Predict the risk factors

Determining the risk factors for each patient before LTx surgery can support clinicians decide to what extent each transplantation is beneficial for each patient. Hence, Delen.A et al. [31] tried to determine predictor variables and risk factors affecting survival time through various machine learning methods (SVM, ANN, MLP, RBF, DT, and K-means for clustering the results). This study identified a group of risk factors and a comprehensive list of predictors in graft survival. Some factors such as age, gender of the recipient, and his medical condition at admission time were discussed in previous studies. While others have been neglected in previous studies such as the recipient’s length of stay after transplantation, and the interaction of gender and ethnicity between the recipient and the donor.

## Predicting quality of life after transplantation

Lung transplantation usually has significant effects on a patient’s health-related quality of life (HRQL). Patients’ satisfaction with the quality of life can affect their physical, emotional health, social, and sexual functioning. Additionally, it aids them to cope with their lives easily [39–41]. One of the main objectives of LTx is to enhance the quality of life in end-stage lung disease in addition to survival rate increment [39]. Thus, Oztekin.A et al. [22] (2018) compared various models like SVM, KNN, and neural networks to predict the quality of life after lung transplantation. The evaluation of three developed models proved that the hybrid GA-SVM model has high performance in comparison to the other two models to predict the quality of life after lung transplantation.

## Different stages of lung transplantation using machine learning methods

**Fig. 4:**
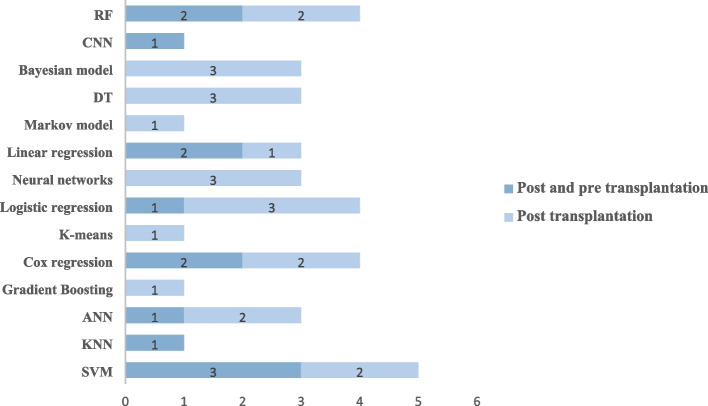
The distribution of articles based on the transplantation phase

The authors utilized the data from donors, candidates, and transplant patients to develop their models. Six studies developed their models based on both pre-and post-transplant patient information [13, 21, 22, 25, 27, 28], and ten studies used only post-transplant patient information to develop their models [19, 20, 23, 24, 26, 29–33]. Two studies used related data from donor and transplant patients [24, 28]. The distribution of articles based on the transplantation phase is shown in Fig. 4.

## Features used in the ML algorithm

Machine learning or data mining algorithms use a series of features as input or predictive factors to build models or classify output variables. These features are selected according to the objective of the researcher to produce the output. Table 4 shows the features used to develop each model and the final goal of creating the model.

Most of the articles (*n* = 12) employed clinical data including demographic data, laboratory data, pulmonary function test results, and follow-up data as input variables, while three articles utilized CT features and images in combination with pulmonary function test results as input variables to create a prediction model. Only one study used CT images to devise a new prediction model [21, 25, 33]. As we can see, the data obtained from pulmonary function tests were the most used in predictive model development.

**Table 4:** Independent variables or features used in machine learning algorithms as input variables

Author	Main objectives	Features	Outcome variable	Data source
Troiani.J et al. [29]	Predict the acute disease events after transplantation	Spirometry variables; values of FEV1 and bronchopulmonary symptoms for these 30 subjects	BOS incidence	A database of home monitoring data
Oztekin.A et al. [30]	Predict survival rate	Events occurring before listing, Recipient angina/cad at registration, Deceased donor-infection pulmonary source, Recipient functional status at registration, Deceased donor-circumstance of death, Recipient age (years), History of cigarette use of the recipient	Survival rate	UNOS
Delen.D et al. [31]	Predict the risk factors for transplantation	socio-demographic, health-related factors about both the donor and the recipients, procedure-related factors, patient follow-up data	Risk factors and patient status after LTx	UNOS
Oztekin.A et al. [32]	Predict survival rate	Recipient’s profile, Match level data, Donor’s profile	Survival rate or transplant success	UNOS
Hosseini-Baharanchi. F et al. [19]	Predict the acute disease events after transplantation	Age at LTX (yr); Type of transplant; Acute rejection episodes; Underlying lung disease; Cytomegalovirus, Death cause	BOS incidence	Post-LTX at the Masih Daneshvari Hospital,
Barbosa.E et al. [33]	Predict the acute disease events after transplantation	PFT data (FEV1, FVC, FEV1/FVC, FEF25-75), baseline CT	BOS incidence	Radiology RIS/PACS data for the period between 06/01/2004 and 06/01/2013
Pande.A et al. [20]	Predict pulmonary functions/ pulmonary symptoms after transplantation	PFT data (FEV1, FVC, FEV1/FVC, FEF25-75) and Age	LTx outcomes	Cleveland Clinic
Barbosa.E et al. [21]	Predict the acute disease events after transplantation	CT features and patient age	BOS incidence	Cardiothoracic clinic
Oztekin.A et al. [22]	Predict quality of life	Donor factors, Recipient factors, Surgical factors, laboratory parameters, hospital stay, intensive care unit (ICU) stay and pulmonary function,	quality of life	UNOS
Mark.E et al. [13]	Predict survival rate	Recipient age, Recipient primary diagnosis, Recipient functional status at transplant, Recipient lung diagnosis grouping, Donor height (cm), Deceased donor history of cigarettes in past	Survival rate	UNOS
Braccioni.F et al. [23]	Predict pulmonary functions/ pulmonary symptoms after transplantation	pulmonary function testing (PFTs), blood gas analysis (ABGs), six-minute walking test (6MWT), and physical examination, DLCO, KCO	Transplantation outcome	tertiary teaching Hospital located in Northeast Italy
Dueñas-Jurado.J et al. [24]	Predict recipient-donor matching	lower pre-transplant carbon dioxide (PCO2) pressure, higher pre-transplant and post-transplant functional vital capacity (FVC), lower donor mechanical ventilation, and shorter ischemia time	Survival rate	Reina Sofı´a university Hospital
Shaish.H et al. [25]	Predict survival rate	HRCT scans	Survival rate	Institutional databases
Stefanuto.P et al. [26]	Predict primary graft dysfunction after lung transplantation	Donor factors, Recipient factors, Surgical factors, Outcomes (1-year Mortality, Ventilation, ICU LOS after Tx, Hospital LOS after TX), Lung function at 3 months	primary graft dysfunction	Harefield Hospital
Su.J et al. [27]	Determine the role of infection in rejection	laboratory parameters, hospital stay, intensive care unit (ICU) stay and pulmonary function,	Rejection with infection	Guangzhou Medical University
Zafar.F et al. [28]	Predict recipient-donor matching	Recipient: Age, Sex, Ethnicity, BMI, Diagnosis, Initial LAS, End LAS, Functional status, eGFR, Albumin, Tobacco use, Infection, Steroid use, ECMO pretransplant, HIV, Recent infection, Ventilation pretransplant, CMV; Donor: Age, Sex, ethnicity, BMI, Tabacco use, Hypertension, Diabetes, Bronchoscopy result, Chest X-Ray result, Pao2/FiO2 ratio, PEEP, Adjusted tidal volume, Arterial blood pH, cause of death, Mechanism of death, CMV, Transplantation characteristics	Matching	UNOS

Despite the high number of features in the dataset, only nine papers applied the feature selection method [13, 20, 22, 23, 26–28, 31]. Feature selection helps researchers select the best set of features to build valuable models of the subjects under study. The applied methods for feature selection include the k-means algorithm (*n* = 1) [31], permutation variable importance (VIMP) (*n* = 1) [20], genetic algorithm (*n* = 1) [22], random forest algorithm (*n* = 3) [13, 23, 27], LASSO Cox regression (*n* = 1) [28], and SVM technique (*n* = 1) [26].

Six of 16 articles developed their models using data sets including more than 1500 samples with more than 30 features [13, 22, 28, 30, 31, 42]. Four studies developed their model using datasets between 1,000 and 100 patients [20, 24, 25, 33], and six studies with less than 100 patients [19, 21, 23, 26, 27, 29]. The sample size varied from 16 to 310,773 individuals.

## The data sources

A source of data or a set of data is needed to develop a model. Data sets are usually large databases of data that are collected and organized for a specific purpose. In terms of data sources, six studies employed the open-source United Network for Organ Sharing (UNOS) dataset to create their models [13, 22, 28, 30–32]. The UNOS dataset is freely available to researchers and has been used in many important linkage studies. UNOS gathers all transplant-related data on every U.S. organ donor, transplant candidate, recipient, and consequence [43]. Other studies have used local databases to build their models [19–21, 23–27, 29, 33]. The data sources with their frequency is described in Table 5.

**Table 5:** Database/Data sources used as data sources in developed models

Data sources	Frequency
UNOS	6
A database of home monitoring data	1
Belgium clinic	1
Cardiothoracic clinic	1
Cleveland Clinic data	1
Guangzhou Medical University	1
Harefield Hospital	1
Institutional Radiology database	1
Masih Daneshvari Hospital database	1
Reina Sofı´a University Hospital	1
Tertiary teaching Hospital located in Northeast Italy	1

## The performance of developed models and evaluation methods

In machine learning, we have to evaluate the stability of developed models to estimate the generalization accuracy of a model’s unseen data. In developing a machine learning model, it is important that the model created is not over-fitting or under-fitting. While underfitting is usually the result of not training the model with enough data, overfitting can have a variety of causes. The ultimate goal of machine learning is to develop a model that performs well with both training data and new data used for predictions. There are two common approaches to evaluating models including creating a holdout dataset or performing cross-validation. Hold-out refers to the strategy in which we divide the data set into a train and a test set. Cross-validation is a technique that involves dividing the original observation data set into a training set that is used to train the model and an independent set that is used to evaluate the analysis. The most common cross-validation method is k-fold cross-validation. In k-fold cross-validation, we can split the input data into k subsets of data. Of 16 articles, 14 articles employed the cross-validation method to detect overfitting and validate the model efficiency. Eight studies utilized a train-test split to evaluate the machine learning algorithm.

**Fig. 5:**
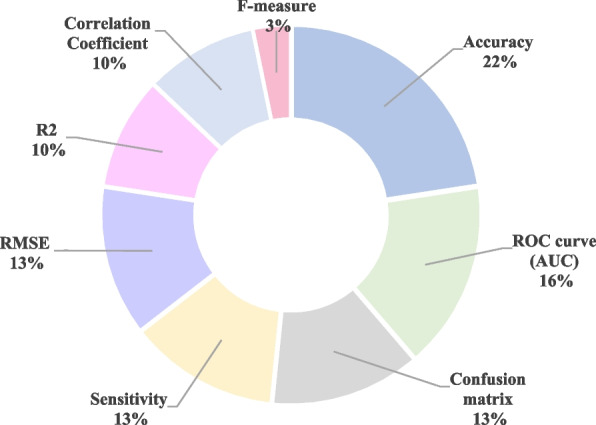
The distribution of applied metrics in reviewed articles

To evaluate the performance of developed models, various metrics were applied including calculating accuracy, sensitivity, F-measure, Root Mean Square Error (RMSE), R-squared (R^2^), correlation matrix, chi-square, or AUC (Area Under the Receiver Operating Characteristics). The most common metric used in studies to evaluate the model alone or to compare it with other developed models was accuracy (*n* = 7). Next, AUC was applied in five studies. The frequency of applied metrics is shown in Fig. 5. The explanation of applied metrics is described in Table 6.

Among seven studies that applied the accuracy metric, five studies this metric in combination with sensitivity and specificity. In one study, the F-measure score was utilized to evaluate the models in combination with accuracy, sensitivity, and specificity.

**Table 6:** The most common metric used in studies

Metrics	Description	Frequency
**Accuracy**	Accuracy is a metric that commonly describes how the developed model performs throughout all datasets.	7 (43.75%)
**Specificity**	Specificity is the extent of true negatives that are accurately anticipated by the developed model.	4 (25%)
**Sensitivity**	Sensitivity could be a degree of how well machine learning demonstrate can distinguish positive instances.	4 (25%)
**F-measure**	The F1-score or F-score may be a degree of a model’s precision on a dataset that can be utilized in classification models.	1 (6.25%)
**Root Mean Square Error (RMSE)**	Root Mean Squared Error (RMSE) and Mean Absolute Error (MAE) are measurements utilized to assess a Regression Model.	4 (25%)
**R-squared**	The R2 score could be a very imperative metric that’s utilized to assess the performance of a regression-based machine learning model. It is known as R squared and is additionally known as the coefficient of assurance.	3 (18.75%)
**AUC or ROC curve**	ROC curve, moreover known as Receiver Operating Characteristics Curve, could be a metric utilized to degree the execution of a classifier model. The ROC curve represents the rate of true positives about the rate of false positives in the classifier model.	5 (31.25%)
**Chi-square or correlation matrix**	A chi-square test is utilized to test the independence of two occasions.	3 (18.75%)
**Confusion matrix**	The confusion matrix is a matrix utilized to show the exact performance of the classification models based on a given set of test data.	4 (25%)

## The quality assessment of reviewed articles

The reviewed articles were evaluated in nine categories suggested by Qiao [18]. The results of the evaluation of articles based on these nine axes are shown in Table 7.

**Table 7:** The results of the quality assessment of reviewed articles

Author	Limits in current non-machine learning approach	Feature engineering	Platforms, packages	Hyperparameters	Valid methods for over-fitting	Stability of results	External data validation	Predictors explanation	Suggested clinical use	Score
Troiani and Carlin [29]	Yes	No	Yes	Yes	No	No	Yes	Yes	Yes	**6**
Oztekin. A et al. [30]	Yes	No	Yes	Yes	Yes	Yes	No	Yes	Yes	**7**
Delen. D et al. [31]	Yes	No	Yes	Yes	Yes	Yes	No	Yes	Yes	**7**
Oztekin. A et al. [32]	Yes	No	Yes	Yes	Yes	Yes	No	No	Yes	**6**
Hosseini-Baharanchi. F et al. [19]	Yes	No	Yes	No	No	Yes	No	Yes	Yes	**5**
Pande. A et al. [20]	Yes	No	Yes	Yes	Yes	No	No	Yes	Yes	**6**
Barbosa. E et al. [33]	Yes	Yes	Yes	No	No	No	No	Yes	Yes	**5**
Oztekin.A et al. [22]	Yes	No	Yes	Yes	No	No	Yes	No	Yes	**5**
Barbosa.E. et al. [21]	Yes	Yes	Yes	Yes	No	Yes	Yes	Yes	Yes	**8**
Mark.E et al. [13]	Yes	Yes	Yes	No	No	Yes	No	Yes	Yes	**6**
Braccioni.F et al. [23]	Yes	No	Yes	Yes	No	Yes	No	Yes	Yes	**6**
Dueñas-Jurado.J et al. [24]	Yes	Yes	Yes	No	No	Yes	No	Yes	Yes	**6**
Shaish.H et al. [25]	Yes	No	Yes	Yes	Yes	Yes	Yes	Yes	Yes	**8**
Zafar.F et al. [28]	Yes	Yes	Yes	No	No	Yes	Yes	Yes	Yes	**7**
Su.J et al. [27]	Yes	Yes	Yes	Yes	No	No	Yes	Yes	Yes	**7**
Stefanuto.P et al. [26]	Yes	Yes	Yes	Yes	Yes	Yes	No	No	Yes	**7**

**Fig. 6:**
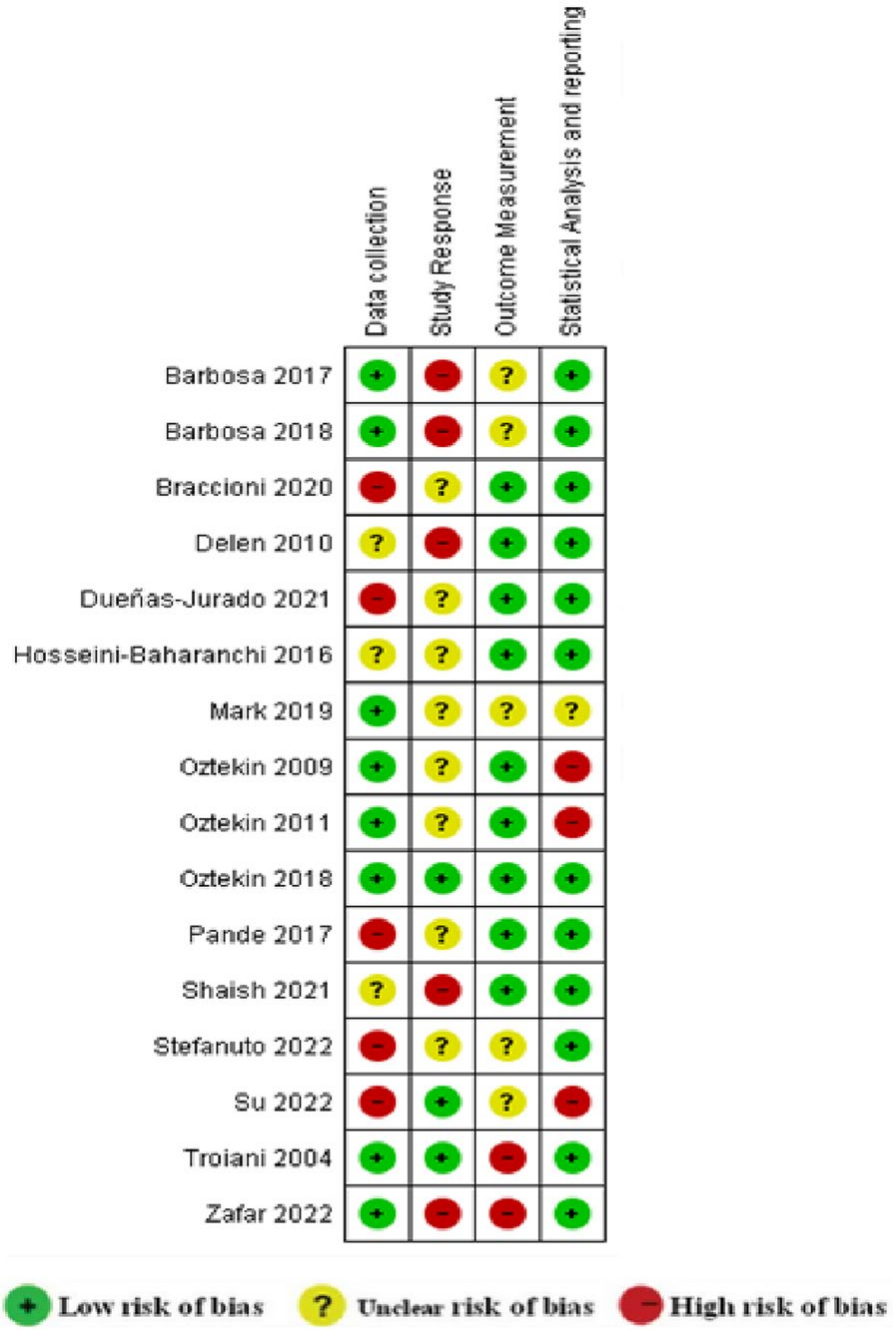
Risk of bias summary regarding each risk of bias item for each included study

The summative score of all articles was above five. The results of the risk of bias assessment in the reviewed articles are shown in Fig 6.

## Discussion

Our systematic review investigated utilizing machine learning in a lung transplant domain. According to the PRISMA checklist, sixteen papers were recognized as eligible articles to identify the usage of machine learning in lung transplantation. Evidence showed that the development of machine learning models in organ transplantation had surprising results in improving transplant success and predicting the likelihood of transplant rejection [15, 44, 45]. Although machine learning techniques can support clinicians predict transplant complications, limited studies have been conducted in lung transplant domains [46].

The majority of the reviewed articles were dedicated to the use of ML models in predicting survival and comorbidities [13, 19, 21, 25, 29, 30, 32, 33]. However, several studies used ML techniques to predict mortality after receiving transplanted organs [44, 45], but no study was done in LTx to predict mortality. Despite the importance of predicting the degree of donor and recipient matching to increase the success of transplant surgery, only two articles were devoted to this topic [24, 28]. Comprehending the benefits of analyzing the main results of LTx with the aid of the machine learning method can make clinicians interested in using this new method based on artificial intelligence.

ML development works best with a high volume of data samples and a rich set of features. Thus, the majority of articles developed their models using UNOS large datasets [47]. Valuable studies have been conducted using UNOS datasets because all of these kinds of data are available for all researchers free of charge [48]. Thus, designing the integrated large database to record all LTx-related data could aid researchers to conduct big data analytics in LTx.

As stated in the results section, most applied techniques were supervised learning methods like SVM, DT, RF, and regression. Although deep learning methods play an essential role in medicine due to their ability to analyze complex data [49], only one study was devoted to deep learning techniques to analyze medical images.

Overfitting is one of the main challenges in supervised ML methods which prevents generalizability [50]. Due to a large amount of LTx data, the authors are faced with high-dimensional data for developing their methods. Usually, all data features are not useful. Hence, feature selection methods to select the best set of features could be effective to design more best-fitting models in medical sciences [51, 52]. Despite all of these benefits, only nine papers applied these kinds of methods. Different metrics have been employed to evaluate the developed ML models. Because the same metrics have not been used to compare the performance of the developed models, it is not possible to compare these techniques. Although the evaluation criteria were mentioned in some studies, the evaluation results are not stated clearly in the articles.

Implementing clinical decision support tools (CDSS) based on developed ML models could support healthcare providers to deliver optimal care to patients [53]. They can analyze a large amount of data in the shortest time and suggest the best treatment options to the physicians [54]. Despite these advantages, no study has designed a decision support system in this field.

This review also faced some limitations. Although search strategies were done in different databases, all machine learning models in this field might not be identified through a literature review. Researchers may have reported only high-performing models. So, publication bias could exist in this review. The author of the studies did not consider all available variables from data sources to develop their models. Considering new variables with the same goals and same data sources might be generated new models with different accuracy. Another limitation of this study is that we considered a wide classification domain of machine learning algorithms in lung transplantation with diverse data sources. Therefore, we cannot compare them in terms of performance. We did not consider any subclassification of any of the considered algorithms or data sources in this study. Ultimately, the development and application of deep learning should be considered the main subject of further study.

## Conclusion

This review showed that applying ML methods could target clinical problems and complications in lung transplantation as one of the complex subjects in medicine. The outcomes of these developed prediction models could aid clinicians to make better and more reliable decisions by extracting new knowledge from the huge volume of data. Deep learning method utilization in lung transplant data analysis could be the main research gap in this field, which can be the main topic of future studies.

## Supplementary information

**Additional file 1.**

## Data Availability

The datasets generated and/or analyzed during the current study are not publicly available but are available from the corresponding author at reasonable request.

## Abbreviations

SVM: Support vector machine
RF: Random Forests
BN: Bayesian network
DT: Decision Tree
KNN: K-nearest neighbors
XGBoost: Gradient Boosting trees
CNN: Convolutional Neural Network
