# Supplementary material for: Machine learning-based techniques to improve lung transplantation outcomes and complications: a systematic review
Source: BMC Med Res Methodol. 2022 Dec 23;22:331. doi: 10.1186/s12874-022-01823-2 (PMC9784000; doi:10.1186/s12874-022-01823-2)
Supplement: Supplementary file 1 — Additional file 1. [file 12874_2022_1823_MOESM1_ESM.docx]

**Appendix A:**

Table A-1- Search strategies in each database

| **Database** | **Search strategies** | **Results (Count of retrevived articles)** |
| --- | --- | --- |
| PubMed | (("Machine learning"[Title/Abstract]) OR ("Machine Learning"[Mesh]) OR ("Deep Learning"[Mesh]) OR ("Deep Learning"[Title/Abstract]) OR ("data mining"[Title/Abstract]) OR ("Neural Network"[Title/Abstract]) OR ("Support vector machine"[Title/Abstract]) OR ("Support Vector Machine"[Mesh]) OR ("Supervised Machine Learning"[Mesh]) OR ("Unsupervised Machine Learning"[Mesh]) OR ("Supervised Machine Learning"[Title/Abstract]) OR ("Unsupervised Machine Learning"[Title/Abstract]) OR ("random forest"[Title/Abstract]) OR ("Bayes Theorem"[Mesh]) OR ("Bayesian network"[Title/Abstract]) OR ("Artificial Neural Network"[Title/Abstract]) OR ("Clustering"[Title/Abstract]) OR ("Decision Tree"[Title/Abstract]) OR ("Decision Trees"[Mesh])) AND (("lung transplantation"[Title/Abstract]) OR ("Lung Transplantation"[Mesh]) OR ("Lung transplant"[Title/Abstract]) OR ("lung transplant recipient"[Title/Abstract]) OR ("lung recipient"[Title/Abstract])) | 101 |
| Scopus | ( ( TITLE-ABS-KEY ( "lung transplant" ) OR TITLE-ABS-KEY ( "lung transplantation" ) OR TITLE-ABS-KEY ( "lung transplant recipient" ) OR TITLE-ABS-KEY ( "lung recipient" ) ) ) AND ( ( TITLE-ABS-KEY ( "Machine learning" ) OR TITLE-ABS-KEY ( "Deep Learning" ) OR TITLE-ABS-KEY ( "data mining" ) OR TITLE-ABS-KEY ( "Neural Network" ) OR TITLE-ABS-KEY ( "Support vector machine" ) OR TITLE-ABS-KEY ( "Supervised Machine Learning" ) OR TITLE-ABS-KEY ( "Unsupervised Machine Learning" ) OR TITLE-ABS-KEY ( "random forest" ) OR TITLE-ABS-KEY ( "Bayes Theorem" ) OR TITLE-ABS-KEY ( "Bayesian network" ) OR TITLE-ABS-KEY ( "Artificial Neural Network" ) OR TITLE-ABS-KEY ( "Clustering" ) OR TITLE-ABS-KEY ( "Decision Tree" ) ) ) | 149 |
| Web of Sciences | 1= "Lung Transplantation" (Topic) or "Lung Transplant" (Topic) or "lung transplant recipient" (Topic) or "lung recipient" (Topic)  2= "Machine learning" (Topic) or "Deep Learning" (Topic) or "data mining" (Topic) or "Neural Network" (Topic) or "Support vector machine" (Topic) or "Supervised Machine Learning" (Topic) or "Unsupervised Machine Learning" (Topic) or "random forest" (Topic) or "Bayes Theorem" (Topic) or "Bayesian network" (Topic) or "Artificial Neural Network" (Topic) or "Clustering" (Topic) or "Decision Tree" (Topic)  (#2) AND (#1) | 93 |
